# Sex Differences in Red Blood Cell Transfusions and 30-Day Mortality in Cardiac Surgery: A Single Center Observational Study

**DOI:** 10.3390/jcm12247674

**Published:** 2023-12-14

**Authors:** Jenni Räsänen, Sten Ellam, Juha Hartikainen, Auni Juutilainen, Jari Halonen

**Affiliations:** 1 Institute of Clinical Medicine, School of Medicine, University of Eastern Finland, 70211 Kuopio, Finland; juha.hartikainen@pshyvinvointialue.fi (J.H.); auni.juutilainen@pshyvinvointialue.fi (A.J.);; 2 Heart Center, Kuopio University Hospital, 70029 Kuopio, Finland; 3 Department of Anesthesiology and Operative Services, Kuopio University Hospital, 70029 Kuopio, Finland; sten.ellam@pshyvinvointialue.fi

**Keywords:** red blood cell transfusion, cardiac surgery, sex difference, 30-day mortality, body mass index, preoperative hemoglobin

## Abstract

In cardiac surgery, women have higher short-term mortality and a higher risk of receiving red blood cell (RBC) transfusions than men. This study’s aim was to evaluate possible sex differences in RBC transfusions in cardiac surgery and their association with preoperative hemoglobin levels, body mass index, and 30-day mortality. A single-center retrospective study was conducted with 1583 patients (1181 men and 402 women) undergoing cardiac surgery. A total of 64.4% of the women and 33.0% of the men received an RBC transfusion. In a multivariable analysis, female sex was an independent predictor of RBC transfusion (OR 3.88, 95% CI 2.95–5.11, *p* < 0.001). Other independent predictors of RBC transfusion were age, preoperative hemoglobin level, and body mass index. The women were more likely to receive RBC transfusions than the men, regardless of the type of cardiac surgery. Decreased transfusion risk was found in all higher-than-normal weight categories in the women, but only in the severe obesity category in the men. Preoperative hemoglobin was similarly associated with RBC transfusion in the men and women. The crude 30-day mortality rate was higher in the women than in the men (2.5% vs. 0.9%, *p* = 0.018). In both sexes, RBC transfusion was associated with an increased probability of death within 30 days.

## 1. Introduction

Perioperative red blood cell (RBC) transfusions have been shown to adversely affect the recovery and prognosis of patients undergoing cardiac surgery, increasing postoperative morbidity and mortality in a dose-dependent manner [1,2,3,4,5,6]. This applies both to short-term and long-term mortality [3,5,6,7,8]. A similar trend has been found not only after cardiac surgery but also after acute coronary syndrome [9,10]. In some studies, a higher impact on mortality has been observed in low-risk patients [6]. Increased use of RBC transfusion has been associated with the female sex [11,12,13], as well as with a multitude of other pre- and perioperative risk factors [14,15]. Despite a decrease in RBC transfusions during recent decades, the female sex has remained an independent predictor of RBC transfusion, as described in a recent study including nearly 19,000 coronary artery bypass grafting (CABG) patients [16].

The role of the female sex is two-fold. Apart from affecting the likelihood of perioperative RBC transfusion, the female sex has been recognized as a risk factor for morbidity and mortality in cardiac surgery [15,17,18,19]. Women have remained at a significantly higher incidence of short-term adverse outcomes following CABG, with no statistically significant improvement in prognosis over the last decade based on a study of 1,297,204 patients [20]. The female sex has also been associated with increased in-hospital mortality following valve surgery, as shown by Bradley et al. in a study including more than 270,000 open cardiac valve operations [21].

The need for sex-specific risk prediction models in cardiac surgery is well recognized [22,23]. Sex is included in the most frequently used risk scores in cardiac surgery, such as the Society of Thoracic Surgeons (STS) mortality score and the European System for Cardiac Operative Risk Evaluation score (EuroSCORE) [24,25]. Sex is also included in four out of five recent RBC transfusion risk prediction models, but not in the risk model for massive blood transfusion [26]. 

Identifying sex-specific risk profiles is important for providing optimal perioperative treatment for both male and female patients scheduled for cardiac surgery. It is unclear whether the excess transfusion of female patients could partly account for their reported worse short-term outcomes. The aim of this study was to evaluate sex differences in RBC transfusions and their association with preoperative hemoglobin, body mass index (BMI), and 30-day mortality. This topic is relevant in the midst of efforts towards more restrictive transfusion practices [27].

## 2. Materials and Methods

### 2.1. Ethics Approval and Informed Consent

This study was approved by the Research Ethics Committee of the Northern Savo Hospital District (No. 1694/13.02.00/2019, 18 December 2019) Informed consent was not required because the study was register-based. This study complies with the Declaration of Helsinki.

### 2.2. Patient Selection

The data were collected retrospectively from a university hospital cardiac surgery database. Patients undergoing off-pump, re-do, and aortic surgery using deep hypothermia procedures were excluded. This study included 1583 nonemergent consecutive patients undergoing CABG, aortic valve replacement (AVR), AVR in combination with CABG, mitral valve repair (MVP) or mitral valve replacement (MVR), MVP/MVR in combination with CABG, and aortic root reconstruction using cardiopulmonary bypass between January 2013 and December 2016.

### 2.3. Anesthetic Management

The anesthetic management was performed according to our hospital protocol, including propofol infusion, sufentanil, and pancuronium boluses. A pulmonary artery catheter was used in the valve and combined procedures. The mean arterial pressure target was above 60 mmHg, and phenylephrine boluses or norepinephrine infusion were used if needed. In patients receiving warfarin treatment, an international normalized ratio (INR) above 1.6 was the trigger for fresh frozen plasma infusion. All patients received 3.0 g of tranexamic acid intravenously. The Hepcon (Medtronic, Minneapolis, MN, USA) protocol was used with an activated coagulation time target of 480 s, and protamine was administered according to the Hepcon calculation. After surgery, the patients were followed in the intensive care unit and were weaned off the ventilator when they fulfilled the following criteria: hemodynamic stability, peripheral temperature of more than 32 °C, cooperativity, and no major bleeding.

### 2.4. Outcomes and Measurements

The primary outcome of this study was the use of RBC transfusion during the first 5 days (the day of operation and four postoperative days). The trigger of RBC transfusion was a hemoglobin level <80 g/L during the perioperative period. The secondary outcome was all-cause mortality within 30 days after the operation. 

BMI was calculated as weight (kg)/height^2^ (m^2^). The patients were divided into four subgroups based on the World Health Organization (WHO) criteria for BMI: normal weight 18.5–24.9 kg/m^2^, overweight 25–29.9 kg/m^2^, obesity class I (mild obesity) 30–34.9 kg/m^2^, and obesity classes II + III (moderate and morbid obesity) ≥35 kg/m^2^.

The preoperative hemoglobin values were divided using sex-specific tertiles, resulting in cut-off limits of 137 g/L and 148 g/L for men and 125 g/L and 135 g/L for women. Preoperative anemia was defined as a hemoglobin concentration <130 g/L for men and <120 g/L for women based on the WHO criteria for anemia.

### 2.5. Statistical Methods

The normality of the distribution of the continuous variables was tested using Kolmogorov–Smirnov and Shapiro–Wilk methods. Since the continuous variables were not normally distributed, they are presented as medians with interquartile ranges, and the categorical variables are expressed as frequencies with percentages. The continuous variables were analyzed using Mann–Whitney U-tests, and the categorical variables were analyzed using chi-squared tests.

A linear regression analysis was applied to evaluate the association between RBC transfusion frequency (%) and BMI (kg/m^2^, whole numbers) and to compare the strength of the association (the steepness of the slopes) between the two sexes. 

A logistic regression analysis was performed using RBC transfusion as the dependent variable. The analyzed predictors were age, sex, preoperative atrial fibrillation, current cigarette smoking, New York Heart Association (NYHA) class, sex-specific hemoglobin tertiles, BMI classes, ejection fraction <40%, arteriosclerosis obliterans, preoperative use of warfarin or low-molecular weight heparin, and glomerular filtration rate (estimated using the Chronic Kidney Disease Epidemiology Collaboration equation). A univariable logistic regression analysis was used to the calculate odds ratio (OR) with a 95% confidence interval (CI) and *p*-value for each potential predictor. A multivariable logistic regression analysis was then performed with predictors for the final model chosen based on the results of univariable analyses (*p* < 0.10) and clinical and theoretical consideration of possible confounding factors. The operation type was forced into the model.

The predicted probability of 30-day mortality as a function of the number of transfused RBC units was derived separately for men and women based on the logistic regression analysis. Sensitivity analyses were performed across subgroups of confounder-adjusted hemoglobin tertiles, BMI classes, and operation types.

*p*-values < 0.05 were considered statistically significant. The analyses were performed using the IBM SPSS statistics 25.0/27.0 software for Windows (SPSS Inc., Chicago, IL, USA).

## 3. Results

### 3.1. Baseline Characteristics

Of the 1583 patients, 1181 (74.6%) were men, as shown in the table for the baseline and perioperative characteristics (Table 1). Almost half of the patients (49.0%) underwent an isolated CABG procedure. The men underwent CABG more often than the women, while aortic valve procedures were more common in the women. Compared with the men, the women were older, more often obese, had lower preoperative hemoglobin, a higher NYHA class, a lower estimated glomerular filtration rate, and they were less often current smokers (all *p*-values < 0.001). No difference was observed in the prevalence of anemia between the sexes.

### 3.2. RBC Transfusions by Sex and Type of Operation

**Figure 1:**
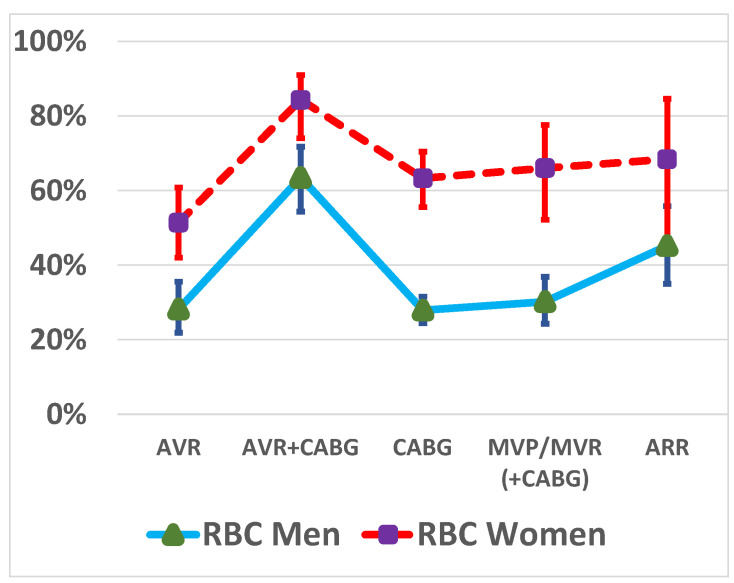
Red blood cell (RBC) transfusion rates by sex and type of operation. CABG, coronary artery bypass grafting; AVR, aortic valve replacement; MVP, mitral valve repair; MVR, mitral valve replacement; ARR, aortic root reconstruction.

A total of 390 (33.0%) men and 259 (64.4%) women received an RBC transfusion (OR for female sex 3.67, 95% CI 2.90–4.66, *p* < 0.001). The women received more RBC transfusions than the men across all operation types: 63.3% vs. 27.9% in CABG (*p* < 0.001), 51.4% vs. 28.2% in AVR (*p* < 0.001), 84.3% vs. 63.5% in AVR + CABG (*p* = 0.002), 66.0% vs. 30.2% in MVP/MVR (+CABG) (*p* < 0.001), and 68.4% vs. 45.2% (*p* = 0.068) in aortic root reconstructions, respectively (Figure 1).

**Figure 2:**
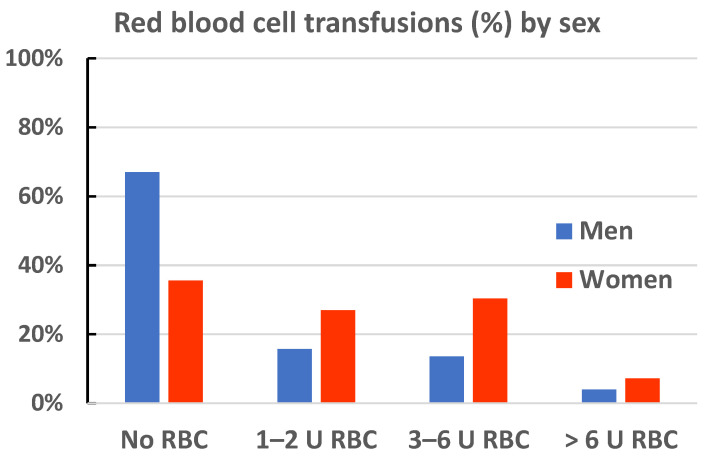
Red blood cell (RBC) transfusion rates by transfusion units in men and women.

The women were overrepresented in the RBC transfusions group, regardless of the number of RBC units transferred (Figure 2).

### 3.3. Linear Regression between BMI and RBC Transfusions by Sex

A linear regression analysis of the association between BMI and RBC transfusions by sex showed a higher Y-intercept in the women than in the men, with statistical significance (*p*-value < 0.001). The regression slope was steeper in the women than in the men (−2.13 vs. −1.15), but not significantly so (two-sided *p*-value, 0.052). In both the men and women, the slope deviated significantly from zero, with two-sided *p*-values < 0.001. 

### 3.4. Univariable Logistic Regression Analysis

In the univariable logistic regression analysis, the significant factors associated with perioperative RBC transfusion in both sexes were age, preoperative hemoglobin levels, preoperative anemia, renal function, and cardiopulmonary bypass time (Table 2). NYHA class 3–4, preoperative atrial fibrillation, peripheral vascular disease, and preoperative use of warfarin were associated with an increased risk of RBC transfusion in men but not in women.

**Figure 3:**
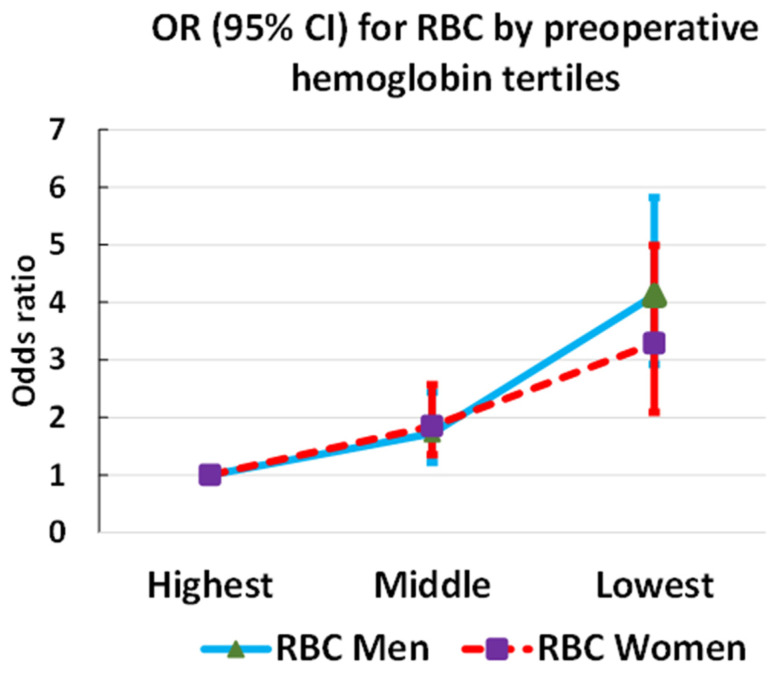
Association of red blood cell (RBC) transfusion with preoperative hemoglobin levels in men and women.

The association of RBC transfusion with sex-specific tertiles of preoperative hemoglobin levels was similar in the men and women (Figure 3, Table 2).

**Figure 4:**
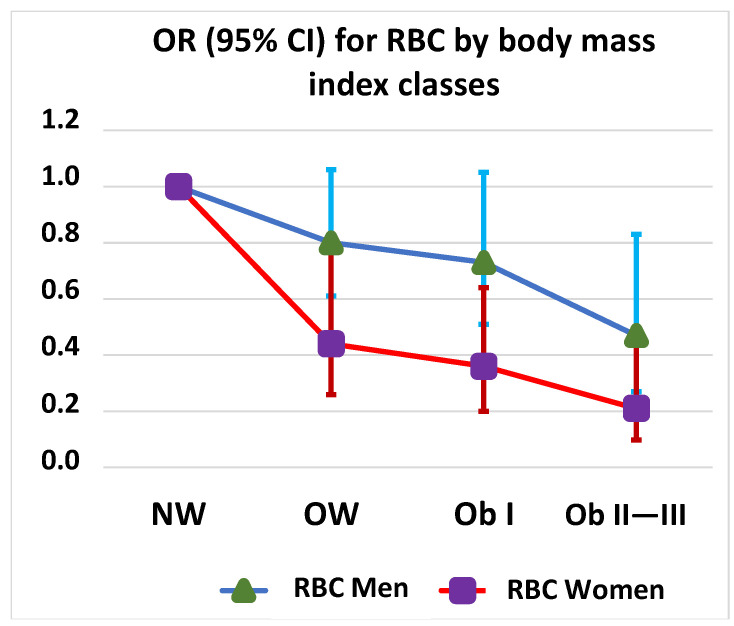
Red blood cell (RBC) transfusion odds ratios (OR) by sex and body mass index class. NW, normal weight; OW, overweight; Ob I, obesity class I; Ob II―III, obesity classes II―III.

Compared with normal weight, overweight, obesity class I, and obesity class II–III were associated with decreased use of RBC transfusion in the women, and a similar albeit weaker trend was observed in the men (statistical significance only between normal weight and obesity class II–III) (Figure 4).

### 3.5. Multivariable Logistic Regression Model

The multivariable logistic regression model included sex, age, BMI class, sex-specific tertiles of preoperative hemoglobin, estimated glomerular filtration rate, NYHA class 3–4 vs. 1–2, and the operation type as explanatory variables. The female sex (OR 3.88, 95% CI 2.95–5.11, *p* < 0.001) proved to be an independent risk factor for RBC transfusion (Table 3). 

The factors that were associated with RBC transfusions in both sexes in the multivariable logistic regression model were age, obesity class II–III compared with normal weight, and preoperative hemoglobin levels. In the women, being overweight and obesity class I compared with a normal weight was also associated with a decreased need for RBC transfusions. In the men, NYHA class 3–4 vs. 1–2 was associated with increased use of RBC transfusion.

### 3.6. Sensitivity Analysis

**Figure 5:**
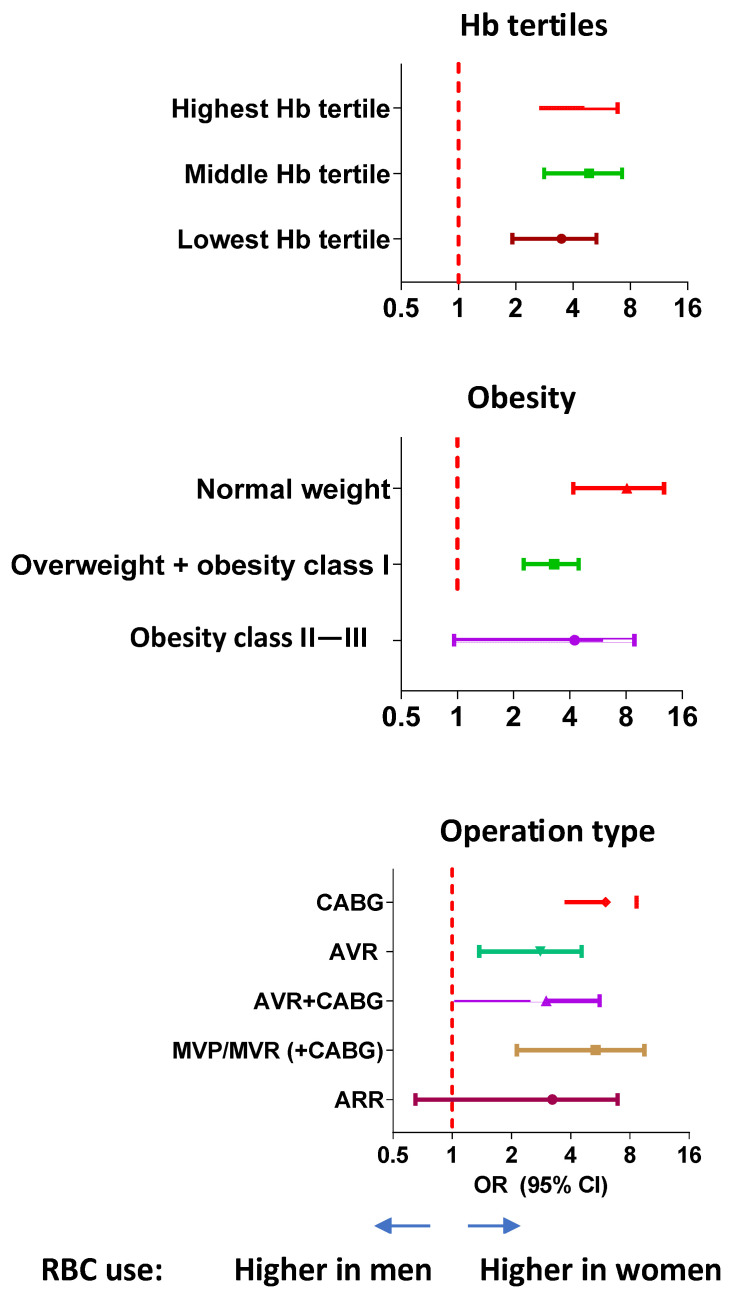
Red blood cell (RBC) transfusions in men and women: sensitivity analysis. The odds ratios with 95% confidence intervals for the association of red blood cell transfusions with the female sex are shown by subgroups of preoperative hemoglobin levels, body mass index class, and type of operation. CABG, coronary artery bypass grafting; AVR, aortic valve replacement; MVP, mitral valve repair; MVR, mitral valve replacement; ARR, aortic root reconstruction.

A sensitivity analysis by subgroups showed that the association of RBC transfusion with the female sex remained significant across sex-specific hemoglobin tertiles, BMI classes except obesity class II + III, and all operation types except aortic root reconstruction (Figure 5).

### 3.7. 30-Day Mortality

**Figure 6:**
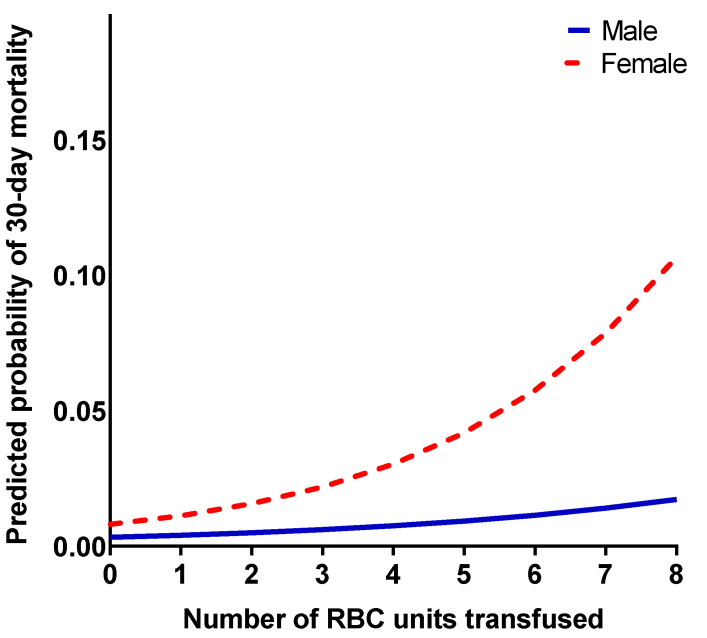
Predicted probability of 30-day mortality (y-axis) by the number of red blood cell units transfused (x-axis). Analyzed separately in men and women.

The overall all-cause 30-day mortality was 1.3% (*n* = 21). The crude mortality rate was higher in the women than in the men (2.5% vs. 0.9%, *p* = 0.018). Most of the patients who died within 30 days had received an RBC transfusion (*n* = 18, 85.7%), with a fully adjusted odds ratio (OR) of 6.16 (95% CI 1.66–22.86, *p* = 0.007). Of the women who died within 30 days, 90.0% had received an RBC transfusion (fully adjusted OR 3.98, 95% CI 0.44–36.12, *p* = 0.220), and of the men, 81.8% had received an RBC transfusion (fully adjusted OR 7.11, 95% CI 1.36–37.18, *p* = 0.002). Figure 6 depicts the sex-specific association of the predicted probability of death within 30 days against the number of RBC units transfused. This shows that although the relative risk estimate (OR) was higher in the men than in the women, the absolute risk of death was higher in the women.

## 4. Discussion

The main result of this study was that women undergoing cardiac surgery were more likely to receive an RBC transfusion than their male counterparts, with prominent differences in all operation types, independent of the number of units transferred and after adjustment for confounding factors. The female risk of RBC transfusion was four-fold, without a decrease after multivariable adjustment. Overweight and obesity decreased the risk of RBC transfusion in women in comparison to normal weight, but in men, the decrease was statistically significant only in patients with severe obesity. Preoperative hemoglobin below the highest tertile was associated with RBC transfusion, the risk below the lowest tertile being three-fold in men and four-fold in women after multivariable adjustment. The association between the number of RBC transfusions and the probability of death within 30 days was evident.

The risk associated with cardiac surgery differs between women and men in two respects. In several but not all studies, outcomes in women have been found to be worse than in men. In addition, more RBC transfusions are given to women than to men, which may indicate an additional factor affecting the unfavorable prognosis in women. In a large study including more than 700,000 cardiac surgery patients, women were 1.7-fold more likely than men to receive allogeneic RBC transfusions [11]. In a study of 3000 CABG patients, the female sex was an independent risk factor for higher use of allogeneic blood product utilization, with an OR of 2.1, which is somewhat lower than in our study [12]. In a study of 24,000 cardiac surgery patients, the female sex was associated with an increased number of transfused units, and each unit of transfused RBC was associated with a 13% higher 30-day mortality [2]. Sex-specific increases per RBC unit were not reported. 

A higher RBC transfusion rate in women has been related to a lower preoperative total blood volume, lower preoperative RBC mass, and larger relative RBC loss [28,29]. According to Stammers et al., women are transfused more often than men, even after having the same hematocrit value. The female sex remained as an independent risk factor for RBC transfusions after adjustment for other independent predictors, such as the total estimated blood volume, volume added during cardiopulmonary bypass, and the volume removed through ultrafiltration [30]. Because clinicians apply the same absolute transfusion thresholds irrespective of a patient’s sex, even though women have a lower baseline RBC volume, women end up receiving more RBC units [30]. This, together with the commonly used liberal transfusion strategy, leads to over-transfusion in women [28].

As both preoperative anemia and RBC transfusion are associated with increased mortality and morbidity and their mutual prognostic role has remained controversial [31], patient blood management requires careful consideration in cardiac surgery. A U-curve was found to describe a safety zone in the context of preoperative anemia and RBC transfusion as a balance between tolerable anemia and corrective interventions [32]. Ripoll et al. found that outcomes in relation to preoperative hemoglobin differed by sex [33], but Tanaka et al. observed no sex difference in the association between preoperative red cell mass and RBC transfusion [34]. In our study, preoperative hemoglobin by sex-specific tertiles showed a similar inverse association with RBC transfusion in men and women, with no sex difference.

Being overweight and obese, which are prevalent cardiovascular risk factors, has paradoxically been found to be associated with a better prognosis than being a normal weight in cardiac surgery—a phenomenon designated as the obesity paradox [35,36]. Being overweight and obese impacts not only mortality but also RBC transfusions, as an increased BMI has been found to be associated with less bleeding and fewer RBC transfusions in patients undergoing cardiac surgery [4,37,38]. We observed a sex difference in the association of being overweight and obese with a reduced need for RBC transfusion, and this association was stronger in women than in men. We could not find any previous studies on sex differences in this association with which to compare our results. 

A large body of evidence has shown a higher mortality in women than in men after cardiac surgery, e.g., CABG [39]. In a study of over 70,000 cardiac surgery patients, the 30-day mortality was 2.7% in women and 1.6% in men, the 1-year mortality was 6.2% in women and 4.1% in men, and the long-term mortality was 19.0% in women and 14.8% in men [23]. For comparison, the 30-day mortality in our study was 2.5% in women and 0.9% in men. In a study of 66,000 patients undergoing isolated CABG or CABG combined with a valve procedure, women had a higher risk of both in-hospital and late mortality than men [40]. Gupta et al. found that women were 32% more likely to die in hospital after CABG and were 25% more likely to be readmitted during the first 90 days after CABG [41]. In a large meta-analysis by Bradley et al., the female sex was associated with a 1.4-fold in-hospital mortality across the spectrum of open-heart valve surgeries [21]. Women undergoing mitral or aortic valve replacement, multiple valve procedures, and valve procedures combined with CABG were at an increased risk compared to men. However, women and men had similar risk in single valve repairs. Mokhles et al. found no sex difference in isolated AVR mortality [22], and Movahed et al. found a persistent reduction in the age-adjusted mortality rate from aortic valve surgery with the elimination of the gender gap in the USA [42]. 

Several explanations have been proposed for worse surgical outcomes in women. Women undergoing cardiac surgery are older, smaller, have more comorbidities, and are more likely to require urgent or emergent operations [19,23]. However, Blankstein et al. found that even after adjusting for age, body surface area, and comorbidities, the female sex was an independent predictor of perioperative mortality [19]. Women more often have non-obstructive coronary artery disease due to coronary artery microvascular dysfunction, while men tend to have obstructive coronary artery disease, which is a more favorable phenotype for revascularization [43,44,45]. However, coronary microvascular dysfunction as the cause of women’s worse prognoses has also been questioned [46,47]. Women more often have atypical symptoms, which can delay diagnosis and treatment [48,49]. There are also some factors related to surgical techniques that do not favor women, such as smaller coronary arteries and underutilization of internal mammary or internal thoracic artery grafts [50]. 

We now raise the question of whether the higher number of RBC transfusions in women in cardiac surgery could be part of the explanation for the worse surgical outcomes observed in women. Although it is somewhat unclear to what extent RBC transfusion simply acts as a marker of a worse prognosis, revealing unadjusted factors, the disadvantages of RBC transfusions are obvious. Trials show that a restrictive transfusion strategy reduces transfused RBC units without increasing mortality or morbidity [27]. Narrowing the gender gap in RBC transfusions would be a much-needed step towards more restrictive blood transfusion practices.

We acknowledge some limitations of our study. The retrospective nature of this single-center study does set inherent limitations for the interpretation of the results. The follow-up of RBC transfusions was 5 days. According to our hospital practice, patients with an uncomplicated recovery were discharged back to the referring hospital on the fourth postoperative day. Therefore, we do not have data on the use of blood products beyond the first 5 days. However, blood products are typically given during the intraoperative period, intensive care treatment, and the first postoperative days. These periods were well covered in our study. We did not have data on perioperative blood loss, the need for resternotomy, or postoperative hemoglobin level as a surrogate of blood loss. The analysis of sex differences in the 30-day mortality was limited by the low mortality rate and the small proportion of women. Although the analyses were adjusted, bias may arise from unknown or unmeasured confounders.

## 5. Conclusions

In conclusion, women undergoing cardiac surgery were more likely to receive an RBC transfusion than their male counterparts, regardless of the operation type. As a new finding, the risk of RBC transfusion decreased with increasing BMI more clearly in women than in men. RBC transfusion was associated with an increased 30-day mortality in the pooled analysis of men and women. Further research is needed to determine whether the number and frequency of RBC transfusions being greater in women than in men is one of the factors that worsens the short-term prognosis of cardiac surgery in women.

## Figures and Tables

**Table 1:** Baseline and perioperative characteristics of the study population. *n* (%) or medians with interquartile ranges.

	Women, *n* = 402	Men, *n* = 1181	*p*-Value
Age	73.0 (65.0–78.0)	66.0 (59.0–74.0)	<0.001
Body mass index	27.6 (24.3–31.6)	27.2 (24.7–30.1)	0.271
Body mass index category ^1^			<0.001
Normal weight	123 (30.6%)	331 (28.0%)	
Overweight	138 (34.3%)	548 (46.4%)	
Obesity Class I	105 (26.1%)	221 (18.7%)	
Obesity Class II–III	36 (9.0%)	81 (6.9%)	
Operation			<0.001
CABG	158 (39.3%)	617 (52.2%)	
AVR	105 (26.1%)	163 (13.8%)	
AVR + CABG	70 (17.4%)	115 (9.7%)	
MVP/MVR (+CABG)	50 (12.4%)	202 (17.1%)	
ARR	19 (4.7%)	84 (7.1%)	
Chronic obstructive pulmonary disease	7 (1.7%)	34 (2.9%)	0.215
Current smoking	32 (8.0%)	171 (14.5%)	< 0.001
Estimated glomerular filtration rate	74.0 (60.3–86.5)	82.6 (70.6–92.7)	<0.001
LVEF < 40%	19 (4.8%)	86 (7.3%)	0.077
NYHA class			<0.001
No symptoms	2 (0.5%)	48 (4.1%)	
1	10 (2.5%)	76 (6.4%)	
2	113 (28.1%)	347 (29.4%)	
3	208 (51.7%)	461 (39.0%)	
4	69 (17.2%)	249 (21.1%)	
Preoperative atrial fibrillation	33 (8.2%)	112 (9.5%)	0.444
Peripheral vascular disease	33 (8.2%)	104 (8.8%)	0.713
Preoperative hemoglobin	130.0 (121.0–139.0)	143.0 (133.0–151.0)	<0.001
Preoperative anemia	88 (21.9%)	229 (19.5%)	0.293
Preoperative LMWH	56 (13.9%)	206 (17.4%)	0.102
Preoperative warfarin	30 (7.5%)	94 (8.0%)	0.749
Cardiopulmonary bypass time, min	105 (82–137)	104 (81–136)	0.726
Red blood cell transfusion			<0.001
0 U	143 (35.6%)	791 (67.0%)	
1–2 U	108 (26.9%)	185 (15.7%)	
3–6 U	122 (30.3%)	159 (13.5%)	
>6 U	29 (7.2%)	46 (3.9%)	

^1^ Body mass index for weight categories: normal weight, 18.5–24.9; overweight, 25–29.9; obesity class I, 30–34.9; obesity class II–III ≥ 35 kg/m^2^. ARR, aortic root reconstruction; AVR, aortic valve replacement; BMI, body mass index; CABG, coronary artery bypass grafting; LMWH, low molecular weight heparin; LVEF, left ventricular ejection fraction; MVP, mitral valve repair; MVR, mitral valve replacement; NYHA, New York Heart Association.

**Table 2:** Univariable logistic regression analyses for predictors of red blood cell transfusions (≥1 U).

	Women		Men		All	
	OR (95% CI)	*p*-Value	OR (95% CI)	*p*-Value	OR (95% CI)	*p*-Value
Female sex					3.67 (2.90–4.66)	< 0.001
Age (10 y)	1.79 (1.42–2.26)	<0.001	1.51 (1.33–1.72)	<0.001	1.74 (1.56–1.94)	<0.001
Body surface area (+0.1)	0.75 (0.67–0.85)	<0.001	0.87 (0.81–0.93)	<0.001	0.76 (0.73–0.80)	<0.001
Body mass index class ^1^						
Normal weight	1 (ref.)		1 (ref.)		1 (ref.)	
Overweight	0.44 (0.26–0.77)	0.004	0.80 (0.61–1.06)	0.124	0.66 (0.52–0.84)	<0.001
Obesity Cl I	0.36 (0.20–0.64)	<0.001	0.73 (0.51–1.05)	0.092	0.68 (0.51–0.90)	0.008
Obesity Cl II–III	0.21 (0.098–0.47)	<0.001	0.47 (0.27–0.83)	0.009	0.43 (0.28–0.66)	<0.001
Preoperative hemoglobin (10 g/L)	0.61 (0.51–0.73)	<0.001	0.62 (0.56–0.68)	<0.001	0.57 (0.53–0.62)	<0.001
Preoperative anemia	4.08 (2.17–7.65)	<0.001	4.63 (3.42–6.28)	<0.001	4.18 (3.21–5.45)	<0.001
Preoperative hemoglobin tertiles ^2^						
Highest	1 (ref.)		1 (ref.)		1 (ref.)	
Middle	2.04 (1.25–3.32)	0.004	1.78 (1.28–2.48)	<0.001	1.76 (1.36–2.28)	<0.001
Lowest	4.35 (2.52–7.51)	<0.001	4.50 (3.27–6.21)	<0.001	3.98 (3.07–5.17)	<0.001
eGFR (10 mL/min/1.73 m^2^)	0.85 (0.75–0.95)	0.006	0.82 (0.77–0.88)	<0.001	0.80 (0.75–0.84)	<0.001
LVEF < 40%	1.23 (0.46–3.30)	0.686	1.09 (0.69–1.73)	0.712	1.00 (0.67–1.50)	0.996
NYHA class 3–4	1.39 (0.90–2.14)	0.142	1.54 (1.20–1.99)	<0.001	1.60 (1.29–1.97)	<0.001
Preoperative atrial fibrillation	0.84 (0.40–1.74)	0.633	1.73 (1.17–2.56)	0.006	1.38 (0.98–1.95)	0.062
Peripheral vascular disease	1.11 (0.52–2.37)	0.779	1.84 (1.23–2.77)	0.003	1.56 (1.10–2.22)	0.012
Cardiopulmonary bypass time (+10 min)	1.16 (1.09–1.23)	<0.001	1.13 (1.09–1.16)	<0.001	1.12 (1.10–1.15)	<0.001
Preoperative LMWH	1.78 (0.94–3.39)	0.078	1.33 (0.97–1.81)	0.074	1.29 (0.99–1.68)	0.062
Preoperative warfarin	0.95 (0.44–2.06)	0.896	2.17 (1.42–3.31)	<0.001	1.71 (1.18–2.47)	0.004

^1^ Compared with normal weight; ^2^ Sex-specific cut-off limits of hemoglobin tertiles: 137 g/L and 148 g/L for men, and 125 g/L and 135 g/L for women. eGFR, estimated glomerular filtration rate; LMWH, low molecular weight heparin; LVEF, left ventricular ejection fraction; NYHA, New York Heart Association.

**Table 3:** Multivariable logistic regression analyses for preoperative predictors of red blood cell transfusions (≥1 U).

	Women		Men		All	
	OR	*p*-Value	OR	*p*-Value	OR	*p*-Value
Female sex					3.88 (2.95–5.11)	<0.001
Age (+10 y)	1.49 (1.12–1.98)	0.006	1.19 (1.01–1.39)	0.034	1.24 (1.08–1.43)	0.002
Body mass index class						
Normal weight	1 (ref.)		1 (ref.)		1 (ref.)	
Overweight	0.43 (0.23–0.79)	0.006	0.84 (0.61–1.15)	0.282	0.72 (0.55–0.95)	0.020
Obesity class I	0.38 (0.20–0.72)	0.003	0.75 (0.50–1.12)	0.165	0.62 (0.44–0.86)	0.005
Obesity class II–III	0.24 (0.10–0.57)	0.001	0.53 (0.28–0.98)	0.044	0.42 (0.25–0.70)	<0.001
Preoperative Hb tertiles ^1^						
Highest tertile	1 (ref.)		1 (ref.)		1 (ref.)	
Middle tertile	1.85 (1.07–3.18)	0.027	1.72 (1.22–2.44)	0.002	1.71 (1.29–2.29)	<0.001
Lowest tertile	3.29 (1.79–6.05)	<0.001	4.12 (2.92–5.82)	<0.001	3.85 (2.86–5.19)	<0.001
eGFR (+10 mL/min/1.73 m^2^)	0.92 (0.80–1.07)	0.299	0.92 (0.85–1.01)	0.067	0.93 (0.86–0.99)	0.041
NYHA 3–4 vs. 1–2	0.94 (0.57–1.57)	0.822	1.41 (1.05–1.90)	0.023	1.25 (0.97–1.61)	0.084
Operation						
CABG	1 (ref.)		1 (ref.)		1 (ref.)	
AVR	0.42 (0.23–0.75)	0.003	0.94 (0.61–1.43)	0.789	0.74 (0.53–1.03)	0.073
AVR + CABG	2.03 (0.93–4.45)	0.077	4.40 (2.79–6.93)	<0.001	3.67 (2.48–5.45)	<0.001
MVP/MVR + (CABG)	0.95 (0.46–1.99)	0.901	1.38 (0.93–2.05)	0.110	1.25 (0.89–1.75)	0.208
ARR	1.53 (0.51–4.59)	0.450	3.67 (2.18–6.17)	<0.001	3.05 (1.90–4.88)	<0.001

^1^ Sex-specific; ARR, aortic root reconstruction; AVR, aortic valve replacement; CABG, coronary artery bypass grafting; eGFR, estimated glomerular filtration rate; Hb, hemoglobin; MVP, mitral valve repair; MVR, mitral valve replacement; NYHA, New York Heart Association class.

## Data Availability

The data cannot be shared for ethical reasons.
